# Two Distinct Modes of Hypoosmotic Medium-Induced Release of Excitatory Amino Acids and Taurine in the Rat Brain *In Vivo*

**DOI:** 10.1371/journal.pone.0003543

**Published:** 2008-10-28

**Authors:** Renée E. Haskew-Layton, Alena Rudkouskaya, Yiqiang Jin, Paul J. Feustel, Harold K. Kimelberg, Alexander A. Mongin

**Affiliations:** 1 Center of Neuropharmacology and Neuroscience, Albany Medical College, Albany, New York, United States of America; 2 Burke Medical Research Institute of Cornell University, White Plains, New York, United States of America; 3 Ordway Research Institute, Albany, New York, United States of America; University of Massachusetts Medical School, United States of America

## Abstract

A variety of physiological and pathological factors induce cellular swelling in the brain. Changes in cell volume activate several types of ion channels, which mediate the release of inorganic and organic osmolytes and allow for compensatory cell volume decrease. Volume-regulated anion channels (VRAC) are thought to be responsible for the release of some of organic osmolytes, including the excitatory neurotransmitters glutamate and aspartate. In the present study, we compared the *in vivo* properties of the swelling-activated release of glutamate, aspartate, and another major brain osmolyte taurine. Cell swelling was induced by perfusion of hypoosmotic (low [NaCl]) medium via a microdialysis probe placed in the rat cortex. The hypoosmotic medium produced several-fold increases in the extracellular levels of glutamate, aspartate and taurine. However, the release of the excitatory amino acids differed from the release of taurine in several respects including: (i) kinetic properties, (ii) sensitivity to isoosmotic changes in [NaCl], and (iii) sensitivity to hydrogen peroxide, which is known to modulate VRAC. Consistent with the involvement of VRAC, hypoosmotic medium-induced release of the excitatory amino acids was inhibited by the anion channel blocker DNDS, but not by the glutamate transporter inhibitor TBOA or Cd^2+^, which inhibits exocytosis. In order to elucidate the mechanisms contributing to taurine release, we studied its release properties in cultured astrocytes and cortical synaptosomes. Similarities between the results obtained *in vivo* and in synaptosomes suggest that the swelling-activated release of taurine *in vivo* may be of neuronal origin. Taken together, our findings indicate that different transport mechanisms and/or distinct cellular sources mediate hypoosmotic medium-induced release of the excitatory amino acids and taurine *in vivo*.

## Introduction

The release of organic osmolytes in response to cellular swelling is mediated by one or more volume-sensitive permeability pathways [1]–[3]. Although this phenomenon occurs in all tissues, it has special significance in the brain since two major swelling-sensitive organic osmolytes, glutamate and taurine, also mediate or modulate neuronal communication [4]–[6]. Several neural pathologies, most notably cerebral ischemia, hyponatremia, hepatic encephalopathy and traumatic brain injury, are associated with pronounced cell swelling, which is largely restricted to astrocytes [7]–[9]. Pathological cell swelling is likely related to tissue damage since pharmacological inhibitors that block volume-sensitive anion permeability pathway(s) suppress the pathological release of the excitatory amino acids, glutamate and aspartate, and reduce infarct size in animal models of stroke and ischemia [10]–[15]. These findings have led to the proposal that the swelling-activated release of excitatory amino acids may play a critical role in promoting ischemic tissue damage [7], [9], [16].

The swelling-induced release of the excitatory amino acids glutamate and aspartate and the sulfonic acid taurine is thought to be mediated by Volume Regulated Anion Channels (VRACs), which are also termed in the literature as Volume Sensitive Outward Rectifying (VSOR) Cl^−^ channels or Volume-Sensitive Organic osmolyte/Anion Channels (VSOAC) [17]–[19]. VRACs are traditionally identified as volume-sensitive Cl^−^/anion channels that are activated in response to cell swelling in nearly all cell types studied. However, in spite of extensive research efforts, the molecular identity of these channels remains unknown [19], [20]. The major physiological role of VRAC is cell volume regulation. Upon activation in swollen cells, VRACs mediate the release of inorganic and organic anions and, in conjunction with swelling-activated K^+^ channels, facilitate reductions in intracellular osmolarity and subsequent regulatory volume decrease. In addition to Cl^−^, VRACs are also permeable to bicarbonate (HCO_3_^−^), several other inorganic anions, as well as small organic osmolytes such as amino acids, polyols, and methylamines [2], [21], [22].

The evidence that taurine release is mediated by VRAC, or a very similar permeability pathway, largely stems from studies in cultured neuronal and glial cells, which show that both swelling-activated [^3^H]taurine and ^125^I^−^ (Cl^−^) fluxes are inhibited by a variety of VRAC blockers, including the selective VRAC inhibitor DCPIB [23]–[27]. Several electrophysiological studies have confirmed that VRACs are permeable to taurine, at least under conditions when its molecule is negatively charged [21], [22], [28]. Nevertheless, there is continuous debate as to whether taurine shares the same permeability pathway with Cl^−^ and other anionic amino acids [29]–[31]. In particular, in several cell types and in brain slices, swelling-activated [^3^H]taurine efflux shows different kinetics and pharmacological properties, when compared to Cl^−^ (^125^I^−^) or D-[^3^H]aspartate release [32]–[37].

Although VRAC and VRAC-mediated amino acid fluxes have been extensively studied in cultured cells, there is limited information regarding their properties in intact brain tissue. Several studies, which used perfusion of hypoosmotic medium via microdialysis probes to induce cell swelling in the brain, found increased extracellular levels of taurine and several amino acids, which are known to permeate through VRAC [38]–[41]. Likewise, Phillis and co-workers found hypoosmotic medium-stimulated release of taurine and the VRAC-permeable amino acids using a cortical cup perfusion technique, and such a release was strongly inhibited by the putative VRAC-blockers DNDS, NPPB, niflumic acid and tamoxifen [42].

In the present work, we used a microdialysis approach in anesthetized animals to compare properties of swelling-activated fluxes of the excitatory amino acids glutamate and aspartate to those of taurine in the rat cortex *in vivo*.

## Materials and Methods

### Animal surgery and microdialysis procedures

All animal procedures performed in this work were approved by the institution's animal care and use committee and adhered to the NIH guidelines for care and use of laboratory animals. Male Sprague-Dawley rats (Taconic Farms), weighing between 325 and 425 g, were allowed free access to food and water. Rats were given atropine sulfate (0.5 mg/kg, i.m.) to reduce respiratory tract fluid secretion, and anesthetized with isoflurane prior to intubation. Intubated rats were mechanically ventilated with a gas mixture of 2.25% isoflurane in 30% O_2_/balance N_2_. A saline drip (0.9% NaCl) was administered intraperitoneally throughout the experimental procedure to prevent dehydration. Body temperature was monitored throughout the experiment with a rectal probe and was maintained between 36°C and 36.5°C with a heating pad.

Animals were placed in a stereotaxic frame and microdialysis probes (2 mm tip, 20 kD cutoff, CMA Microdialysis, North Chelmsford, MA, U.S.A.) were slowly lowered through burr holes into the frontoparietal cortex (from bregma, 1 mm anterior; ±4 mm lateral; 2.6 mm down from the dura). Artificial cerebral spinal fluid (aCSF; in mM: 120 NaCl, 2.7 KCl, 1 MgSO_4_, 1.2 CaCl_2_, 25 NaHCO_3_, 0.05 ascorbic acid; pH = 7.3) was perfused at 2 µl/min through the microdialysis probes. After two hours of probe stabilization at least two 20 minute perfusate samples were collected by a CMA-170 refrigerated fraction collector (CMA Microdialysis) to determine baseline amino acid levels before the application of drug or hypoosmotic medium. Hypoosmotic aCSF (in mM: 25 NaCl, 2.7 KCl, 1 MgSO_4_, 1.2 CaCl_2_, 25 NaHCO_3_, 0.05 ascorbic acid; pH = 7.3) was perfused at 2 µl/min for one hour and perfusate samples were collected every 5 minutes. Each rat was implanted with two microdialysis probes placed bilaterally in the cortex, with one probe serving as a control (hypoosmotic solution only) and the probe on the other side (chosen at random) serving as the experimental condition (hypoosmotic solution plus drug). All drugs were delivered through the microdialysis probes.

### Amino acid analysis in microdialysate samples

Dialysate concentrations of the amino acids were determined by reverse-phase high performance liquid chromatography (HPLC) using a Hewlett-Packard Series 1100 HPLC system. Pre-column derivatization of the amino acids was done with o-phthaldialdehyde/2-mercaptoethanol. The derivatives were separated using a C18 Varian column (4.6×100 mm, 3 µm particle diameter). The fluorescence signal was detected by a Hewlett-Packard 1046A programmable fluorescence detector. Amino acid standards were used to calculate the concentrations of the amino acids in the perfusate.

### Preparation of primary astrocyte cultures

Confluent primary astrocyte cultures were prepared from the cerebral cortex of newborn Sprague-Dawley rats as described elsewhere [43], with minor modifications summarized below. Newborn Sprague-Dawley rats were euthanized by rapid decapitation, the cerebral cortices were separated from the meninges and basal ganglia, and tissue was dissociated using the neutral protease Dispase II (Roche Applied Science, Indianapolis, IN, U.S.A.). Dissociated cells were seeded on poly-D-lysine coated 18×18 mm glass coverslips (Caroline Biological Supply Co, Burlington, NC, U.S.A.) for efflux experiments, or 12-well tissue culture plates for uptake experiments. Cell cultures were grown for 3–4 weeks in Minimal Essential Medium (MEM) supplemented with 10% heat inactivated horse serum (HIHS), 50 U/ml penicillin and 50 µg/ml streptomycin at 37°C in a humidified atmosphere of 5% CO_2_/95% air. Culture medium was replaced twice a week. After two weeks of cultivation, penicillin and streptomycin were removed from the culture medium. Immunocytochemistry showed ≥95% of the cells stained positively for the astrocytic marker glial fibrillary acid protein.

### Preparation of rat cortical synaptosomes

Rat cortical synaptosomes were isolated from the cortical tissue of male Sprague-Dawley rats (Taconic Farms) weighing between 180 and 230 g according to [44] with modifications described elsewhere [45]. Final synaptosomal pellets were resuspended in HEPES-buffered medium containing (in mM): 135 NaCl, 3.8 KCl, 1.2 MgSO_4_, 1.3 CaCl_2_, 1.2 KH_2_PO_4_, 10 D-glucose, 10 HEPES; pH = 7.4. Synaptosomes were incubated for 30–40 min at 37°C in order to allow them to restore transmembrane ion gradients before further use in amino acid release experiments.

### [^3^H]Taurine and D-[^3^H]aspartate efflux assays

[^3^H]Taurine or D-[^3^H]aspartate efflux measurements were performed in astrocyte cultures as follows. Astrocytes grown on glass coverslips were loaded overnight with either [^3^H]taurine (4 µCi/ml) or D-[^3^H]aspartate (4 µCi/ml) in 2.5 ml of MEM containing 10% HIHS in a CO_2_ incubator set for 5% CO_2_/95% air at 37°C. Before the start of the efflux measurements, the cells were washed free of extracellular isotope and residual serum-containing medium in HEPES-buffered solution. The basal HEPES-buffered medium contained (in mM): 135 NaCl, 3.8 KCl, 1.2 MgSO_4_, 1.3 CaCl_2_, 1.2 KH_2_PO_4_, 10 D-glucose, 10 HEPES; pH = 7.4. The coverslips were inserted into a Lucite perfusion chamber which had a depression precisely cut in the bottom to accommodate the coverslip and a Teflon screw top leaving a space above the cells of around 100–150 µm in height. The cells were superfused at a flow rate of 1.2 ml/min in an incubator set at 37°C with isoosmotic or hypoosmotic HEPES-buffered media. To prepare hypoosmotic medium, the concentration of NaCl was reduced to 85 mM. The osmolarities of all buffers were checked using a freezing point osmometer (µOsmette, Precision Systems, Natick, MA, U.S.A.) and were 287–290 and 197–200 mOsm for isoosmotic and hypoosmotic media, respectively. Superfusate fractions were collected at one minute intervals. At the end of each experiment, the isotope remaining in the cells was extracted with a solution containing 2% sodium dodecyl sulfate (SDS) plus 8 mM EDTA. Four ml Ecoscint scintillation cocktail (National Diagnostics, Atlanta, GA, U.S.A.) was added and each fraction was counted for [^3^H] in a Tri-Carb 1900TR Liquid Scintillation Analyzer (PerkinElmer, Boston, MA, U.S.A.). Percent fractional isotope release for each time point was calculated by dividing radioactivity released in each 1-min interval by the radioactivity left in the cells (the sum of all the radioactive counts in the remaining fractions up to the beginning of the fraction being measured, plus the radioactivity left in the cell digest).

In a few experiments, astrocytes were simultaneously loaded with D-[^3^H]aspartate (2 µCi/ml) and [^14^C]taurine (1 µCi/ml) to compare properties of swelling-activated fluxes of excitatory amino acids and taurine in one cell preparation. In these instances [^3^H] and [^14^C] radioactivity was determined in the same perfusate samples using a Tri-Carb 1900TR Liquid Scintillation Analyzer and double-label DPM software.

To measure taurine release in synaptosomal preparations, synaptosomal suspensions were loaded with [^3^H]taurine (0.5 µCi/ml) for 1 hour at 37°C in basal HEPES-buffered solution. The extracellular isotope was washed by adding 9 volumes of ice-cold medium containing (in mM): 243 sucrose, 5 KCl, 1.2 MgSO_4_, 10 HEPES, 10 glucose; pH = 7.4. Synaptosomes were sedimented (10,000 g, 2 min at 2°C) and resuspended in the same sucrose medium, which prevents spontaneous synaptosome depolarization at low temperatures. Aliquots of [^3^H]taurine-loaded synaptosomes (∼0.2–0.3 mg protein) were injected in glass tubes containing 4.5 mL of HEPES-buffered basal, low [NaCl] hypoosmotic, or low [NaCl] isoosmotic media, as specified in the Results section. After 5-min incubation at 37°C, taurine efflux was terminated by rapid vacuum filtration through GF/C glass microfiber filters (Whatman-GE Healthcare, Florham Park, NJ, U.S.A.). Filters were placed in scintillation vials containing a 4 ml Ecoscint scintillation cocktail and counted for radioactivity remaining in the synaptosomes. Relative taurine efflux values (% loaded/5 min) were calculated by comparing the radioactivity in experimental samples to isotope content in samples filtered through GF/C without incubation at 37°C (“0 time”).

### [^3^H]Taurine and D-[^3^H]aspartate uptake assay

Cultured astrocytes for these experiments were grown in 12-well tissue culture plates according to the cell culture method described in the previous section. Serum-containing medium was washed out, and the cells were incubated for 30 minutes at 37°C with basal HEPES-buffered medium containing 0.5 µCi/mL of [^3^H]taurine or D-[^3^H]aspartate plus 10 µM of unlabeled taurine or L-glutamate, respectively. Following the 30-min incubation period cells were washed four times with ice-cold physiological phosphate buffered solution. Cells were then lysed with 2% SDS plus 8 mM EDTA. The isotope content in the lysate was used as a measure of taurine ([^3^H]taurine) or L-glutamate (D-[^3^H]aspartate) uptake. Four ml Ecoscint scintillation cocktail was added to each lysates and [^3^H] was counted in a Liquid Scintillation Analyzer.

### Statistical analysis

The statistical significance of the differences in the amino acid release and uptake were determined with ANOVA or repeated measures ANOVA, as specified throughout the text and in figure legends. For the *in vivo* experiments, planned comparisons were performed with repeated measures ANOVA to determine differences in amino acid release only during hypoosmotic medium exposure. Origin 7.5 (OriginLab, Northampton, MA) and Statistica 6.1 (StatSoft, Tulsa, OH) were used for statistical analysis.

### Chemicals

Cadmium chloride (CdCl_2_), hydrogen peroxide (H_2_O_2_), mannitol and ouabain were purchased from Sigma (St. Louis, MI, U.S.A). [^3^H]Taurine or D-[^3^H]aspartate were from GE Healthcare-Amersham (Buckinghamshire, U.K.). DL-Threo-β-benzyloxyaspartic acid (DL-TBOA) was obtained from Tocris (Ellisville, MI, U.S.A.). 4,4′-dinitrostilbene-2,2′-disulfonic acid, disodium salt (DNDS) and all cell culture reagents were from Invitrogen (Carlsbad, CA, U.S.A.). All other chemicals including amino acid standards for the HPLC experiments were purchased from Sigma or Aldrich (Milwaukee, WI, U.S.A.) and were the highest purity available.

## Results

### Differences in kinetics of cortical amino acid and taurine release in response to perfusion of hypoosmotic medium or low NaCl isoosmotic medium

**Figure 1:**
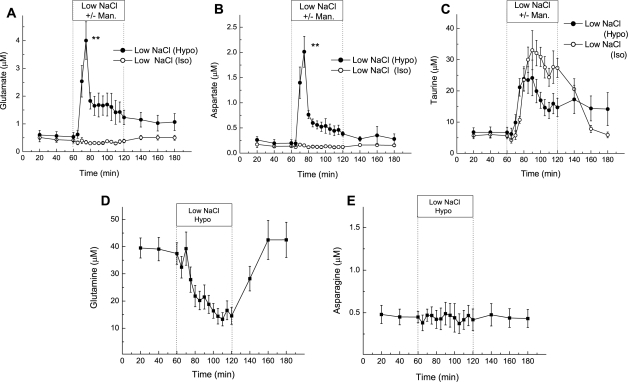
Effect of hypoosmotic or isoosmotic low [NaCl] medium on amino acid levels measured in the rat cortex *in vivo*. (a–c) Microdialysis probes, implanted in the rat frontoparietal cortex, were perfused with hypoosmotic medium (−95 mM NaCl, −65% osmolarity) or isoosmotic low NaCl medium (−95 mM NaCl +167 mM mannitol) for one hour. In these experiments, the rat brain was perfused with both the hypoosmotic and isoosmotic medium on opposite sides of the cortex. The data represent average dialysate levels of glutamate (a), aspartate (b), and taurine (c) ±SEM from 5 rats. ** p<0.01, hypoosmotic vs. isoosmotic low [NaCl], repeated measures ANOVA. (d–e) In several experiments dialysate levels of glutamine (d, N = 5), and asparagine (e, n = 3) were additionally measured on the “hypoosmotic” side of the brain.

In order to examine volume-sensitive amino acid release *in vivo*, rat cortices were perfused via a microdialysis probe with hypoosmotic medium in which [NaCl] was reduced from 120 to 25 mM NaCl (65% reduction in osmolarity; medium also contained 25 mM NaHCO_3_ and other salts as specified in the Methods section). We used a larger reduction in medium osmolarity compared to that typically employed *in vitro* to account for the fact hypoosmotic media perfused via microdialysis probes are gradually diluted with the extracellular fluids upon their diffusion in the brain. Hypoosmotic medium initiated substantial increases in the levels of VRAC-permeable glutamate, aspartate and taurine (Fig. 1a–c). In the same experiments the extracellular levels of the VRAC-impermeable amino acids, asparagine and glutamine, were either downregulated (glutamine) or not altered (asparagine) by the hypoosmotic medium (Fig. 1d–e). Increases in the extracellular levels of glutamate and aspartate had similar kinetics. Dialysate levels of both amino acids peaked at 15 minutes (∼6.5- and ∼5-fold increases over baseline, for glutamate and aspartate, respectively), then quickly decreased to levels which were only 2-3-fold higher than the basal release, with additional recovery observed after switching to isoosmotic medium (Fig. 1a, b). In contrast, in the same samples, the swelling-activated release of taurine was consistently delayed by 5 minutes versus excitatory amino acids, had a substantially slower inactivation, and never recovered after returning to isoosmotic conditions (Fig. 1c).

To determine if the increases in glutamate, aspartate and taurine levels were due to changes in osmolarity or a consequence of reduced [NaCl]_e_, the same rats were simultaneously perfused, via microdialysis probes placed in the contralateral cortex, with low [NaCl] (25 mM) medium that was made isoosmotic by the addition of 167 mM mannitol. The isoosmotic low [NaCl] medium failed to induce an increase in dialysate concentrations of glutamate and aspartate (Fig 1a, b). In the same samples, however, we found a large increase in the extracellular levels of taurine in response to isoosmotic [NaCl]_e_ reduction, which was not statistically different from the hypoosmotic-stimulated augmentation (Fig 1c).

**Figure 2:**
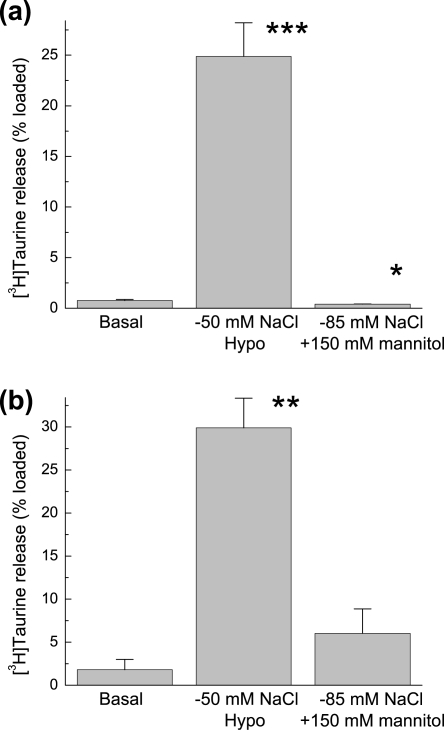
Isoosmotic low [NaCl] medium does not induce taurine release from cultured rat astrocytes but modestly enhances taurine release from rat cortical synaptosomes. (a) Effect of hypoosmotic or isoosmotic reductions in [NaCl] on [^3^H]taurine release from astrocytes. The data represent mean values ±SEM of integral 10-min releases under isoosmotic (Basal), hypoosmotic (Hypo), or isoosmotic low [NaCl] solutions. n = 4 for each group. *p<0.05, ***p<0.001, vs. basal. (b) Integral 5-min releases of [^3^H]taurine from synaptosomes exposed to isoosmotic (Basal), hypoosmotic (Hypo) or isoosmotic media with lowered [NaCl]. Means ±SEM of 3 experiments. ** p<0.01, vs. basal.

In order to understand the nature of the low [NaCl]-induced taurine release, we exposed cultured astrocytes and isolated nerve endings (synaptosomes) prepared from rat cortical tissue to low [NaCl] media made isoosmotic with mannitol. In striking contrast to the *in vivo* microdialysis data, cultured astrocytes preloaded with [^3^H]taurine failed to show any increase in taurine release levels when perfused with the same low [NaCl] isoosmotic medium (Fig 2a). In cortical synaptosomes, we found modest (∼3-fold) increases in [^3^H]taurine release under isoosmotic low [NaCI] conditions (2b). However, such increases were much smaller when compared to the releases induced by the hypoosmotic reduction in [NaCI] (∼15 fold, Fig. 2b). This was in contrast to our *in vivo* data which showed very similar increases in taurine levels with both hypoosmotic and isoosmotic low [NaCl] medium (compare Figs 2b and 1c).

**Figure 3:**
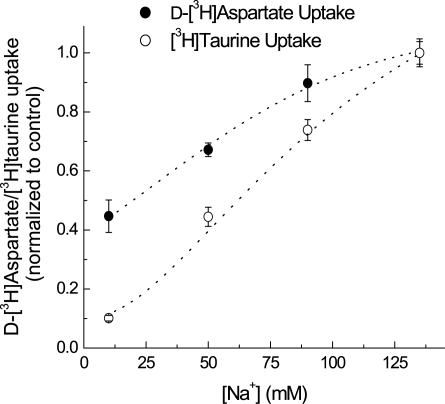
Dependence of taurine and glutamate uptake on extracellular [Na^+^] in cultured astrocytes. Taurine and glutamate transport rates were measured in primary astrocyte cultures using [^3^H]taurine and d-[^3^H]aspartate. Extracellular concentrations of amino acids were adjusted to 10 µM using unlabeled taurine or l-glutamate. To compare glutamate versus taurine uptake, the values were normalized to uptake levels under basal conditions ([Na^+^]_o_ = 135 mM). Note that under basal conditions absolute d-[^3^H]aspartate uptake rate (nmols/mg protein) was ∼5-fold higher compared to taurine. Data are the mean values ±SEM of three experiments from each group.

Since taurine transporter function is dependent on the transmembrane Na^+^ gradient, we speculated that the increased levels of taurine seen *in vivo* upon application of low extracellular [NaCl] hypoosmotic or isoosmotic media may in part be due to inhibition of the taurine transporter. To address this issue, we evaluated how decreases in [Na^+^]_e_ affect [^3^H]taurine uptake in cultured astrocytes. Given that glutamate and aspartate levels *in vivo* are insensitive to the isoosmotic decrease of [Na^+^]_e_ (see Fig. 1a, b), we additionally compared the effect of low [Na^+^]_e_ on [^3^H]taurine uptake to the uptake of D-[^3^H]aspartate. As seen in Fig. 3, decreases in [Na^+^]_e_ to 50 mM (equivalent to the low [Na^+^]_e_ used in the *in vivo* experiments) significantly inhibited both [^3^H]taurine and D-[^3^H]aspartate (L-glutamate) uptake, with taurine uptake inhibited to a greater extent. However, the observed *in vitro* difference in the transporters' sensitivities to [Na^+^]_e_ may not in itself be sufficient to explain the drastic sensitivity of taurine release to isoosmotic modulation of the [NaCl] observed *in vivo*.

### Effects of the anion channel blocker DNDS on hypoosmotic medium-stimulated amino acid and taurine release *in vivo* and *in vitro*

**Figure 4:**
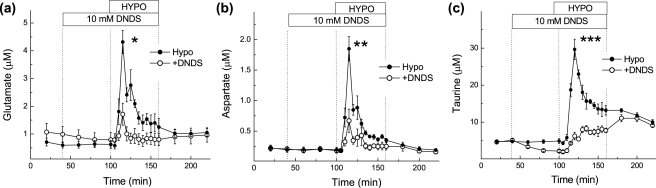
Effect of the anion channel blocker DNDS on hypoosmotic medium-induced amino acid release in the rat cortex. (a–c) Microdialysis probes were perfused on opposite sides of the cortex with hypoosmotic medium (HYPO, −65% osmolarity) in the presence or absence of 10 mM DNDS, given one hour prior to and during one-hour hypoosmotic medium perfusion. The data represent average dialysate levels of glutamate (a), aspartate (b) and taurine (c) +/−SEM from 5 rats. * p<0.05, HYPO vs. HYPO+DNDS (glutamate); ** p<0.01, HYPO vs. HYPO+DNDS (aspartate); *** p<0.001 HYPO vs. HYPO+DNDS (taurine). Significance was determined by repeated measures ANOVA.

Given that our data show that taurine and excitatory amino acid levels are differentially regulated *in vivo* by low [NaCl], we further investigated the potential mechanisms responsible for the elevated amino acid levels in response to hypoosmotic medium by using different amino acid transport inhibitors. Our first aim was to determine if the increases in extracellular excitatory amino acid levels is mediated by a VRAC-like pathway, as has been extensively shown *in vitro*. Therefore, we used the anion channel blocker DNDS, which has an IC_50_ for VRACs of ∼1–2 mM [46]. Although DNDS has a low potency for inhibiting VRACs, it is one of the few anion channel inhibitors that can be used in microdialysis studies because it does not produce toxic effects or changes in the basal amino acid release levels. The more potent VRAC blockers NPPB or phloretin caused strong and progressive increases in microdialysate glutamate and aspartate levels, which likely reflect cytotoxicity (Y. Jin, R.E. Haskew-Layton, P.J. Feustel, H.K. Kimelberg, A.A. Mongin, unpublished observations). Ten mM DNDS, when added one hour prior to and during hypoosmotic medium exposure, significantly inhibited the hypoosmotic-stimulated release of the excitatory amino acids glutamate and aspartate, but did not alter basal excitatory amino acid levels (Fig 4a, b). DNDS also potently inhibited hypoosmotic taurine release, and in addition, also reduced taurine levels under basal conditions (Fig. 4c).

**Figure 5:**
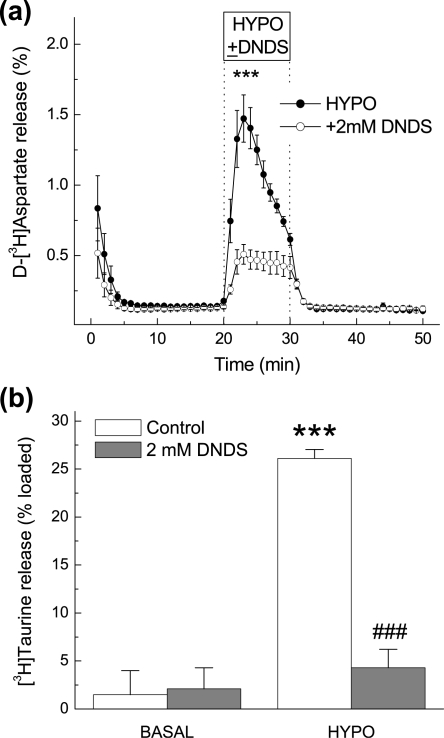
Effect of DNDS on swelling-activated D-[^3^H]aspartate release from cultured astrocytes and swelling-activated [^3^H]taurine uptake in cortical synaptosomes. (a) Cultured astrocytes preloaded with D-[^3^H]aspartate were superfused with hypoosmotic medium in the presence or absence of 2 mM DNDS. The data are the mean values of five experiments for each group ±SEM. *** p<0.001 hypo, vs. DNDS. (b) Release of preloaded [^3^H]taurine from cortical synaptosomes was measured under isoosmotic (BASAL) and hypoosmotic (HYPO) conditions in the presence or absence of 2 mM DNDS. The data are the mean values of integral 10-min [^3^H]taurine release ±SEM of three experiments performed in quadruplicate. ***p<0.001 vs. isoosmotic control (BASAL), ^###^p<0.001 vs. hypoosmotic control (HYPO).

To verify the efficacy of DNDS as a VRAC blocker we tested the effect of DNDS on swelling-activated D-[^3^H]aspartate release in cultured astrocytes, which is entirely mediated by VRACs [47]. Two mM DNDS was sufficient to suppress D-[^3^H]aspartate release by ∼70% (Fig. 5a), was similarly effective against astrocytic [^3^H]taurine release (∼75%, data not shown), and nearly completely suppressed swelling-activated [^3^H]taurine release from cortical synaptosomes (Fig. 5b). To further explore whether DNDS may inhibit taurine transporters and in this way affects extracellular taurine levels *in vivo*, we tested the effects of DNDS on taurine uptake in cultured astrocytes. DNDS did not affect astrocytic [^3^H]taurine uptake up to the concentration of 32 mM, suggesting that this compound does not alter taurine transporter function (data not shown).

### Effects of the Ca^2+^ channel blocker Cd^2+^ on hypoosmotic medium-stimulated amino acid and taurine release *in vivo*

**Figure 6:**
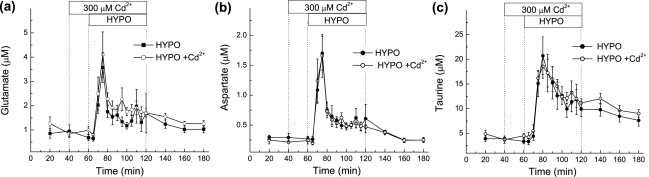
Effect of the Ca^2+^ channel blocker Cd^2+^ on hypoosmotic medium-induced amino acid release in the cortex. (a–c) Microdialysis probes were perfused with hypoosmotic medium (HYPO) in the presence or absence of 300 µM Cd^2+^ given 20 minutes prior to and during one-hour hypoosmotic medium perfusion. Each rat had two microdialysis probes implanted on opposite sides of the cortex (one perfused with HYPO alone and the other with HYPO+Cd^2+^). The data represent the average dialysate levels ±SEM of glutamate (a), aspartate (b), and taurine (c) from 5 rats.

To additionally explore the mechanisms responsible for the excitatory amino acid release *in vivo*, we tested for the contribution of alternative release mechanisms. One such mechanism is synaptic Ca^2+^-dependent release from a vesicular pool. Several reports suggest that hypoosmotic medium induces membrane depolarization and promotes increases in [Ca^2+^]_i_ in synaptosomes and brain slices, which may trigger exocytotic neurotransmitter release [48]–[50]. To exclude the possible involvement of exocytosis in mediating hypoosmotic-induced amino acid release we used a broad spectrum blocker of voltage-sensitive Ca^2+^ channels, cadmium (Cd^2+^). Ca^2+^-free artificial cerebral spinal fluid containing 300 µM Cd^2+^, given 20 minutes prior to and during Ca^2+^-free hypoosmotic medium perfusion, did not alter glutamate, aspartate or taurine release (Fig. 6a–c). Previous studies have found that 300 µM Cd^2+^, administered through microdialysis probes, is effective in blocking electrically stimulated serotonin release and membrane depolarization-induced norepinephrine release [51], [52], and 30 µM Cd^2+^ is sufficient to reduce tonic glutamate release in the amygdala [53]. Thus, our data showing that amino acid release *in vivo* is insensitive to 300 µM Cd^2+^ suggests that hypoosmotic-stimulated excitatory amino acid release is not due to the stimulation of exocytosis.

### Effects of the glutamate transporter blocker DL-TBOA on hypoosmotic medium-stimulated excitatory amino acid release *in vivo*

**Figure 7:**
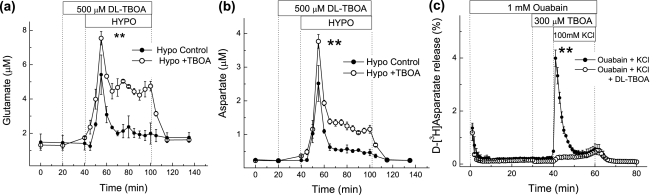
Effect of the glutamate transporter inhibitor dl-TBOA on hypoosmotic medium induced amino acid release in the cortex and glutamate transporter reversal in cultured astrocytes. (a–b) Microdialysis probes implanted on opposite sides of the cortex were perfused with hypoosmotic medium in the presence or absence of 500 µM dl-TBOA, given 20 minutes prior to and during one hour hypoosmotic medium perfusion. The data represent average dialysate levels of glutamate (a), aspartate (b) ±SEM from 4 rats. ** p<0.01 HYPO vs. HYPO+TBOA. (c) DL-TBOA effectively prevented reversal of glutamate transporter in cultured astrocytes. Cultured astrocytes were superfused for one hour with 1 mM ouabain and additionally for 20 min high [KCl] (100 mM) plus ouabain to induce glutamate transporter reversal. 300 µM dl-TBOA was given 10 minutes prior to and during the high [KCl] perfusion in the presence of ouabain. The data are the average values ±SEM for three experiments in each group. ** p<0.01 KCl vs. KCl+TBOA.

Low [Na^+^]_e_ may potentially elevate dialysate glutamate and aspartate levels by inducing the reversal of glutamate transporters, which are dependent on normal Na^+^ and K^+^ gradients [54]. Therefore to rule out the involvement of glutamate transport reversal in mediating hypoosmotic-stimulated excitatory amino acid release we used the broad-spectrum non-transportable glutamate transporter blocker DL-TBOA that blocks all neuronal and glial transporters [55], [54]. 500 µM DL-TBOA given 20 minutes prior to and during hypoosmotic medium perfusion, significantly increased rather than decreased excitatory amino acid release and did not affect taurine levels (Fig. 7a, b), suggesting that normal transport operation is maintained under hypoosmotic conditions and transport reversal is not responsible for the hypoosmotic medium-stimulated excitatory amino acid release. As expected, the *in vivo* extracellular levels of taurine were not affected by DL-TBOA (data not shown).

Although DL-TBOA has been well characterized as an inhibitor of normal glutamate transporter function [55], we wanted to verify DL-TBOA's effectiveness in blocking glutamate transporters working in the reverse mode. Cultured astrocytes, preloaded with D-[^3^H]aspartate, were treated for 40 minutes with 1.0 mM ouabain to increase [Na^+^]_i_, prior to and during 20 minutes perfusion with an isoosmotic 100 mM [K^+^]_e_ medium. These treatment conditions have previously been shown to induce the reversal of glutamate transporters in cultured astrocytes [56]. *In vitro*, 300 µM DL-TBOA given 10 minutes prior to and during 10 mM [K^+^] perfusion completely blocked glutamate transport reversal-induced D-[^3^H]aspartate release, verifying that DL-TBOA is an effective inhibitor of glutamate transport reversal (Fig. 7c).

### Effects of H_2_O_2_ on hypoosmotic-stimulated release of excitatory amino acid and taurine *in vivo* and *in vitro*

**Figure 8:**
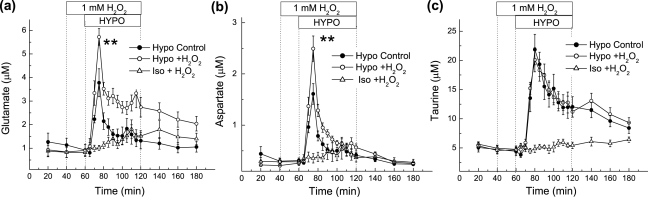
Effect of H_2_O_2_ on hypoosmotic medium induced amino acid release in the cortex. (a–c) Two microdialysis probes implanted on opposite sides of the cortex were perfused with hypoosmotic medium in the presence or absence of 1 mM H_2_O_2_ given 20 minutes prior to and during one-hour hypoosmotic medium perfusion. The data represent the average dialysate levels ±SEM of glutamate (a), aspartate (b) and taurine (c) from 9 rats. ** p<0.01 HYPO vs. HYPO+H_2_O_2_. In separate experiments, rats were perfused with 1 mM H_2_O_2_ alone (N = 5).

We further investigated if reactive oxygen species modulate swelling-sensitive excitatory amino acid release in the brain, as seen in cultured astrocytes (Haskew-Layton et al., 2005). One mM H_2_O_2_, administered 20 minutes prior to and during hypoosmotic medium perfusion, did not affect basal levels of glutamate or aspartate but significantly enhanced the swelling-evoked release of both excitatory amino acids (Fig. 8a,b). In contrast, H_2_O_2_ did not alter dialysate levels of taurine under hypoosmotic conditions (Fig. 8c), suggesting that excitatory amino acids and taurine release are differentially regulated. To verify that H_2_O_2_ does not upregulate glutamate and aspartate release via a VRAC-independent mechanism, we tested the effects of 1 mM H_2_O_2_ on amino acid levels in the absence of hypoosmotic medium in a separate set of experiments. As seen in Fig. 8a, b, when superfused under isoosmotic conditions, 1 mM H_2_O_2_ did not produce a substantial increase in excitatory amino acid levels but did cause a small gradual upward shift in the baseline.

Since H_2_O_2_ has been reported to alter glutamate uptake [57], we tested the effects of 1–1,000 µM H_2_O_2_ on the excitatory amino acid uptake in cultured astrocytes. H_2_O_2_ did not alter the excitatory amino acid uptake *in vitro* up to the highest concentration tested (data not shown). These data, in conjunction with the lack of a H_2_O_2_ effect on the basal levels of excitatory amino acid *in vivo*, suggest that the effects of H_2_O_2_ on hypoosmotic glutamate and aspartate levels are unlikely due to uptake inhibition.

**Figure 9:**
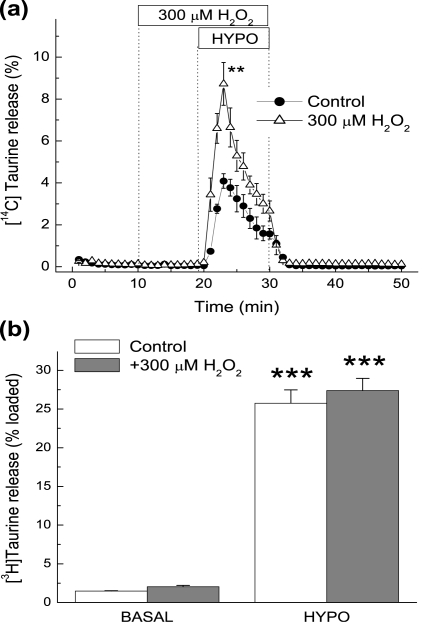
Effect of H_2_O_2_ on swelling-activated taurine release from cultured astrocytes and cortical synaptosomes. (a) The effect of H_2_O_2_ on swelling-activated [^14^C]taurine release from cultured astrocytes. Astrocytes were preloaded overnight with [^14^C]taurine and D-[^3^H]aspartate. 300 µM H_2_O_2_ was added to the media 10 min before and during exposure to hypoosmotic medium. For clarity, only [^14^C]taurine release is shown. The data are the mean values ±SEM of three experiments. **p<0.01 vs. hypotonic control. (b) The effect of H_2_O_2_ on swelling activated [^3^H]taurine release from rat cortical synaptosomes. Integral [^3^H]taurine release was measured for 5 minutes as described in Material and methods. 300 µM H_2_O_2_ was present in media 10 min before and during measurements of taurine release. Data are the mean values ±SEM of three independent experiments performed in quadruplicate. ***p<0.001 vs. basal release.

In order to model the effects of H_2_O_2_ on taurine release in astroglial and neuronal cells, we tested *in vitro* the effect of 300 µM H_2_O_2_ on swelling-activated amino acid release in cultured rat astrocytes and cortical synaptosomes. Astrocytes were simultaneously preloaded with D-[^3^H]aspartate and [^14^C]taurine to reveal any potential differences between release properties of the excitatory amino acids and taurine in the same cells. As in our previous study (Haskew-Layton et al., 2005), H_2_O_2_ strongly potentiated the swelling-induced release of D-[^3^H]aspartate (data not shown), as well as the release of taurine (Fig. 9a) by 2-3-fold. In striking contrast, the same concentration of H_2_O_2_ was completely ineffective in potentiating [^3^H]taurine release in synaptosomes, whether H_2_O_2_ added 10 minutes before and during application of hypoosmotic medium (Fig. 9b) or acutely (data not shown). These results suggest that the H_2_O_2_-insensitive, hypoosmotic-medium stimulated taurine release observed *in vivo* may originate from a neuronal compartment.

## Discussion

In the present *in vivo* study we employed a microdialysis approach to compare properties of the swelling-activated release of major organic osmolytes, the excitatory amino acids glutamate and aspartate and the sulfonic acid taurine, in the rat cortex. Swelling-activated amino acid release may mimic pathological processes that occur in ischemia and several other neuropathologies, where extensive astroglial cell swelling has been detected [7], [9]. To model the effects of cell swelling without the contribution of numerous volume-insensitive glutamate release pathways, we utilized perfusion of hypoosmotic medium via a microdialysis probe, rather than performing the studies in the ischemic brain. The major finding of our work is that hypoosmotic-medium induced release of excitatory amino acids and taurine exhibit significant differences in their kinetic properties, sensitivity to isoosmotic changes in extracellular [NaCl] and response to perfusion of the reactive oxygen species H_2_O_2_. Such differences indicate that these organic osmolytes are released from different cellular pools and/or via different release pathways.

### Properties of hypoosmotic medium-induced excitatory amino acid release are consistent with the involvement of VRAC

Previous findings from the literature suggest that there is substantial similarity between swelling-activated amino acid release *in vitro* and *in situ*. In cultured astrocytes and neuronal cells, hypoosmotic medium promotes cell swelling and triggers the release of several uncharged or negatively charged amino acids such as glycine, alanine, taurine, glutamate, and aspartate [23], [24], [26], [47], [58], [59]. Several *in situ* studies performed in brain slices have found that the release properties of isotope-labeled and endogenous excitatory amino acids and taurine are similar to those observed *in vitro* [36], [60]–[62]. Such organic osmolyte release occurs via a non-saturable pathway, which is inhibited by a variety of Cl^−^ channel blockers, and therefore likely mediated by an anion channel. *In vitro* electrophysiological studies found that volume-regulated anion channels (VRAC) are permeable to glutamate, aspartate, taurine, and glycine, but not to the majority of other amino acids [21], [22], [63]–[65]. However, it is currently debated whether one or more permeability pathways contribute to the release of organic osmolytes [29]–[31]. Furthermore, some reports additionally proposed that hypoosmotic swelling may promote the release of excitatory amino acids via a Ca^2+^-independent mode of exocytosis [66], [67].

*In vivo*, our present work and several previous studies found that hypoosmotic medium stimulates the release of VRAC-permeable amino acids (glutamate, aspartate, and taurine), while the levels of VRAC-impermeable amino acids (*e.g.*, asparagine and glutamine) remain unaffected or decreased [38], [39], [42]. The broad spectrum Cl^−^ channel blocker DNDS inhibited the release of excitatory amino acids and taurine, at concentrations that block VRAC activity *in vitro*. We have found that DNDS is well tolerated *in vivo*, unlike other commonly used and more potent VRAC blockers, such as NPPB and phloretin. Consistent with the involvement of VRAC, increases in the extracellular levels of the excitatory amino acids were seen upon application of hypoosmotic medium (low [NaCl]_e_) but not in response to isoosmotic changes in [NaCl]_e_ (NaCl replaced with mannitol). Furthermore, swelling-induced excitatory amino acid release was not blocked by inhibitors of two alternative glutamate and aspartate release pathways, *i.e.* reversal of glutamate transporters and exocytotic release.

Although cell swelling is thought to be the primary stimulus responsible for initiating VRAC opening in the pathological brain, little else is known about the activation or modulation of VRACs in the intact tissue. Our recent *in vitro* work demonstrated that swelling activated excitatory amino acid release via VRAC is potently modulated by reactive nitrogen species and reactive oxygen species [68], [69]. In the present microdialysis experiments, the reactive oxygen species H_2_O_2_ strongly increased hypoosmotic levels of glutamate and aspartate, but had little effect when administered under isoosmotic conditions. Taken together with the pharmacological data, these findings are in line with the idea that VRAC is the primary source of the excitatory amino acid release in response to hypoosmotic medium-induced (and pathological) cell swelling.

### Hypoosmotic medium-induced release of taurine differs from the excitatory amino acid release

Unexpectedly, we found marked differences in taurine and excitatory amino acid release. Taurine is widely regarded as an important osmoregulatory molecule in the brain and in other tissues because it is one of the most abundant organic osmolytes and effectively permeates a putative VRAC-like pathway [23], [22], [28], [43], [70]. Consistent with its osmoregulatory role, taurine release in cultured astrocytes, hippocampal slices, and *in vivo* microdialysis experiments has been found to be potently upregulated under hypoosmotic conditions (reductions in [NaCl]_e_), but is insensitive to isoosmotic changes in [NaCl]_e_ [23], [41], [66]. Therefore, our observation, that cortical taurine levels are strongly elevated by either hypoosmotic or isoosmotic low [NaCl] media, was rather surprising. Nevertheless, such a finding is not unique. A similar sensitivity of taurine to changes in [NaCl]_e_ independent of changes in osmolarity has been found in two microdialysis studies measuring taurine levels in the rat hippocampus and in slices prepared from the mouse brain stem [71], [72]. Using an alternative approach, in which extracellular amino acids were sampled via a cortical cup, Phillis *et al.* also observed that replacement of extracellular NaCl with choline-Cl or MMDG-Cl strongly elevated superfusate levels of taurine but not those of glutamate or aspartate [73].

Besides the differences in sensitivity to isoosmotic [NaCl]_e_ decreases, taurine release was (i) consistently delayed by ∼5 minutes, compared to the swelling-activate release of the excitatory amino acids, (ii) showed much slower inactivation, and (iii) was completely insensitive to the application of H_2_O_2_. Since the amino acid measurements were performed in the same samples, these data unequivocally point to different release mechanisms or different cellular sources. The idea of diverse transport pathways for taurine and other osmolytes has been suggested in the past, based on dissimilar properties of taurine and excitatory amino acid release in cultured astrocytes, hippocampal brain slices, and in a mammary cell line [35], [66], [74]. However, because the molecular identity of the volume-sensitive organic osmolyte release pathway is unknown, this hypothesis is difficult to address.

An alternative hypothesis that would explain the atypical behavior of taurine is that in the cortex taurine and excitatory amino acids are released from different cellular pools. Glutamate is uniformly distributed in the brain, however it is somewhat more concentrated in neurons since its concentration in astrocytes is lowered by the activity of glutamine synthase [75]. In contrast, the cellular localization of taurine is highly heterogeneous. Depending on the brain region, taurine is concentrated within either glial cells or neurons. For instance, in the cerebellum and the putamen taurine is primarily localized to neurons, while in the thalamus, hypothalamus and brain stem it is concentrated in glial cells [76]–[78]. In the cortex there have been conflicting reports suggesting that taurine is preferentially localized to either neurons or glial cells [79], [80].

**Figure 10:**
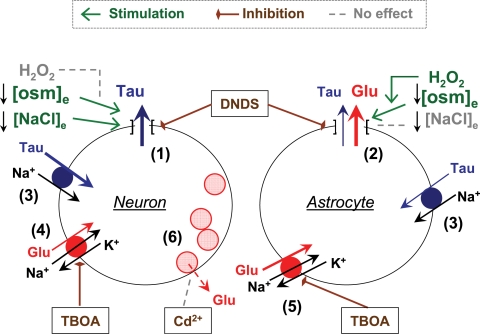
Hypothetical explanation of the experimental data showing differences in taurine and glutamate release *in vivo*. A reduction in medium osmolarity (↓[osm]_e_) in the rat cortex causes an increase in the extracellular levels of the excitatory amino acids glutamate and aspartate and the sulfonic acid taurine via a mechanism sensitive to the anion channel blocker DNDS. Despite these similarities, the excitatory amino acid and taurine release demonstrate different kinetics and are likely mediated by different transport pathways (1 and 2) and/or originate from different cellular sources. The taurine pathway (1) but not the excitatory amino acid pathway (2) is activated by isoosmotic lowering of [NaCl]_e_. Conversely, the swelling-activated excitatory amino acid release pathway (2) but not the taurine pathway (1) is potentiated by H_2_O_2_. Alternative transport pathways that were considered in this study include: [Na^+^]_e_-dependent taurine transporters (3), [Na^+^]_e_/[K^+^]_i_-dependent glutamate transporters in neurons (4), and in astrocytes (5), which are sensitive to TBOA; and vesicular glutamate release (6), which is sensitive to the voltage-gated Ca^2+^ channel blocker Cd^2+^. Based on the similarities of excitatory amino acid release *in vivo* and in cultured astrocytes, we speculate that glutamate release *in vivo* largely originates from glial cells. Similarities between taurine release *in vivo* and in synaptosomes suggest that taurine release may be of a neuronal origin.

In order to model properties of astroglial and neuronal taurine release we performed experiments in primary rat astrocytes and cortical synaptosomes. In cultured astrocytes, taurine release was completely dissimilar to the release *in vivo*: it was absolutely insensitive to isoosmotic decreases in [NaCl]_e_, and was strongly potentiated by H_2_O_2_ in swollen cells. Since we were unable to mimic the astrocytic profile of taurine release *in vivo*, it suggests that astrocytes may contribute to only a negligible portion of taurine release in the cortex. On the other hand, similar to our *in vivo* data, swelling-activated taurine release in synaptosomes mimicked the *in vivo* release in that it was completely insensitive to H_2_O_2_ and sensitive to isoosmotic reductions in [NaCl]_e_ (albeit to a much weaker degree than the *in vivo* response). Although these synaptosomal data do not perfectly match the *in vivo* results, they suggest a possibility that taurine release in the cerebral cortex originates from a neuronal pool. Ineffective uptake of extracellular taurine *in vivo* under hypoosmotic conditions and in response to isoosmotic reductions in [NaCl] may be determined by a lower density of taurine transporters and their high dependence on extracellular [Na^+^] and [Cl^−^] (see Fig. 3). In contrast, glutamate transporters are expressed at a very high density in the brain and driven by the transmembrane gradients of K^+^, Na^+^, and Cl^−^, and are therefore less sensitive to changes in [NaCl]_e_ [54], [75]. An integrated model, providing an explanation for our *in vivo* and *in vitro* data, is presented in Fig. 10.

### Relevance to pathophysiological amino acid and taurine release in ischemia

There is ample evidence that VRAC contributes to pathological excitatory amino acid release in a number of neurological conditions associated with cell swelling, including ischemia, hyponatremia, hepatic encephalopathy, and traumatic brain injury (reviewed in [7], [9]). In animal ischemia models, VRAC blockers reduce pathological elevations in the extracellular levels of excitatory amino acids [10], [11], [14], [81], and potently protect the animal brain against ischemic damage [12], [82], [83]. Most recently, the selective VRAC inhibitor DCPIB has been found to reduce intra-ischemic glutamate release and potently reduce ischemic infarction in a rat focal reversible ischemia model [15]. Consistent with *in vivo* findings, in a slice model of cerebral spreading depression, VRAC inhibitors delay the onset of glutamate-dependent depolarizations and reduce glutamate release, which is at least partially associated with cell swelling [84]. Interestingly, characteristics of intra-ischemic taurine release also show strong deviation from those of glutamate and aspartate. For instance, in a rat global ischemia model ischemic striatal taurine release was weekly sensitive to 1 and 10 mM DNDS, as compared to the strong inhibition of pathological excitatory amino acid release [11]. Although in the present experiments, both the hypoosmotic release of taurine and the excitatory amino acids were potently suppressed by DNDS, the global ischemia data are consistent with the idea that taurine and excitatory amino acids may be released from different cellular pools and/or via different transport mechanisms. Properties of swelling activated excitatory amino acid release *in vivo* strongly resembled the positive modulation of the excitatory amino acid release via a putative VRAC pathway, and in particular were strongly upregulated by H_2_O_2_, as seen in cultured astrocytes [69]. Since H_2_O_2_ levels are strongly upregulated in ischemia and reperfusion [85], [86], the additive effects of cell swelling and reactive oxygen species may contribute to excitotoxic tissue damage in stroke and perhaps other neuropathologies.

In summary, we found that cellular swelling in the rat cortex *in vivo* triggers release of glutamate and aspartate with properties strongly resembling swelling-activated excitatory amino acid release in cultured astrocytes, which is thought to be mediated by VRAC [47]. In contrast, hypoosmotic medium-induced taurine release seemingly is derived from a different cellular source or mediated by different transporter mechanism(s). Since taurine release properties could be mimicked in synaptosomal preparations, we speculate that such release may originate from a neuronal compartment.
